# Ulinastatin May Significantly Improve Postoperative Cognitive Function of Elderly Patients Undergoing Spinal Surgery by Reducing the Translocation of Lipopolysaccharide and Systemic Inflammation

**DOI:** 10.3389/fphar.2018.01007

**Published:** 2018-10-09

**Authors:** Min Zhang, Yan-Hua Zhang, Hui-Qun Fu, Qing-Ming Zhang, Tian-Long Wang

**Affiliations:** ^1^Department of Anesthesiology, Xuanwu Hospital, Capital Medical University, Beijing, China; ^2^Department of Orthopedics, Xuanwu Hospital, Capital Medical University, Beijing, China

**Keywords:** ulinastatin, elderly patients, spine surgery, lipopolysaccharide, systemic inflammation, POCD

## Abstract

**Background:** Studies have shown that perioperative inflammatory response is one of the important factors that caused postoperative cognitive dysfunction (POCD). Ulinastatin is a broad-spectrum protease inhibitor that inhibits inflammatory. We investigated the effects of ulinastatin on inflammatory response and early postoperative cognitive function in elderly patients undergoing spinal surgery.

**Methods:** This clinical trial was approved by the Xuanwu Hospital Ethical Committee (Registration number: ChiCTR-IPR-16008931). Sixty elderly patients undergoing elective spinal surgery with American Society of Anesthesiologists (ASA) status of I–II were randomized into ulinastatin and control groups; total intravenous anesthesia was performed. The elderly patients in ulinastatin group underwent intravenous infusion of ulinastatin 10,000 units/kg following anesthesia induction and before surgical incision, and 5000 units/kg on post-operative days 1 and 2. Cognitive function was determined with Montreal Cognitive Assessment (MOCA) test preoperatively and on post-operative day 7 by a neurologist. Serum lipopolysaccharide (LPS), interleukin-6 (IL-6), C-reactive protein (CRP), and matrix metalloprotease-9 (MMP-9) concentration levels were measured at baseline, the end of surgery, and on post-operative days 1 and 3.

**Results:** All elderly patients completed the study. Ulinastatin infusion significantly reduced the incidence of POCD in elderly patients undergoing spine surgery (ulinastatin group 16% vs. control group 43%, *χ*^2^ = 5.079, *P* = 0.024, *P* < 0.05). The elderly patients in ulinastatin group exhibited lower serum LPS, IL-6, CRP, and MMP-9 concentrations, as well as a shortened peak value duration, compared with those in the control group following surgery (*P* < 0.05).

**Conclusion:** Systemic inflammation and translocation of LPS were inhibited by the infusion of ulinastatin in elderly patients undergoing spinal surgery. The anti-inflammation intervention with ulinastatin can significantly improve the elderly patients’ postoperative cognitive function.

## Introduction

The mechanism of postoperative cognitive dysfunction (POCD) in elderly individuals is related to excessive systemic inflammation caused by surgical trauma in elderly individuals, as well as an imbalance in the circulatory and central nervous system’s immune responses (increased pro-inflammatory substances and reduced anti-inflammatory substances) (Nathan and Rodney, 2008; Hu et al., 2010). Perioperative alarms include the release of high mobility group protein (HMGB1), neutrophils, and monocyte cytoplasmic proteins (S100A8 and S100A9) from sterile wound tissue (Lu et al., 2015; Susana and Mervyn, 2016). These can stimulate the systemic inflammatory response, which plays a key role in the occurrence and development of POCD. Anti-inflammatory and anti-HMGB1 treatment can improve the occurrence of POCD (Rasmussen, 2006). Abdominal surgery can lead to destruction of the intestinal barrier, such that endotoxin (lipopolysaccharide, LPS) are absorbed into the blood where it can stimulate the systemic inflammatory response (Schietroma et al., 2013). However, the relationship between LPS levels, systemic inflammatory responses triggered by non-abdominal surgery, and POCD has not yet been elucidated.

Organic inflammatory response can promote the generation of MMP-9. MMP-9 can resolve and destroy the basement membrane of BBB (Blood Brain Barrier), may change the cerebral vascular permeability and cause the BBB impairment (Hu et al., 2012). BBB permeability changes can promote a large number of circulatory system inflammatory mediators around the central nervous system, a direct attack on neuronal cells or indirect activation of microglial cells, resulting in a series of physiological and pathological processes, and eventually resulting in abnormalities in nerve cells, as well as clinical manifestations of postoperative short-term cognitive decline (Bruno et al., 2009).

Ulinastatin is a urinary trypsin inhibitor (UTI) derived from human urine, which can inhibit enzyme activity and effectively reduce systemic inflammatory response (Atal and Atal, 2016). UTI, a serine protease inhibitor, has been widely used for patients with acute inflammatory disorders such as disseminated intravascular coagulation, shock, and pancreatitis (Inoue et al., 2005). Ulinastatin inhibits systemic inflammatory responses and reduces inflammatory factor expression and metalloproteinase activation. Concomitantly, ulinastatin can inhibit enzymatic activity and stabilize lysosomal membranes, such as matrix metalloprotease-9 (MMP-9) (Li et al., 2018), trypsin, granulocytic elastase, and cathepsin (Inoue et al., 2009; Lee et al., 2010; Fang et al., 2011). Through this mechanism, ulinastatin can protect intestinal barrier function. In addition, ulinastatin inhibits neutrophil and monocyte activation, preventing the release of inflammatory factors (Lee et al., 2010). However, whether ulinastatin can inhibit LPS and inflammatory factors triggered by non-abdominal surgery and improve cognitive function has not been elucidated. Therefore, the aim of this study was to determine the effects of ulinastatin on the inflammatory response and early POCD in elderly patients undergoing spinal surgery.

## Materials and Methods

### Patients and the Controls

This study was a prospective, randomized, double-blind trial approved by the Xuanwu Hospital Ethical Committee (Registration number: ChiCTR-IPR-16008931); written informed consent was obtained from each patient. In this study, we enrolled 60 patients undergoing lumbar discectomy with general anesthesia and 30 age-matched control volunteers to examine the correlation between ulinastatin and serum lipopolysaccharide (LPS), MMP-9, interleukin-6 (IL-6), and C-reactive protein (CRP) levels with early POCD in patients.

We enrolled 60 patients older than 65 years (age ≤ 85 years) with ASA status of I-II. Thirty community volunteers older than 65 years (age ≤ 85 years) were examined to exclude the practice effect of repeated neuropsychological testing. Exclusion criteria were: Mini-mental State Examination (MMSE) score ≤23, acute or chronic infectious diseases or trauma, tumor, taking anti-inflammatory drugs or immunosuppressants, WBC ≥ 10000 × 10^9^/L before surgery, a stroke in the prior 6 months or any other central nervous system diseases, lower digestive diseases, severe deafness or vision problems, illiteracy, and/or communication difficulties related to pronunciation or dialect and postoperative delirium, and refusal or unexpected discharge. We interviewed the patients on the day before surgery and collected baseline data, including age, sex, gender, body-mass index (BMI), past medical history, education history, and MMSE and Montreal Cognitive Assessment (MOCA) score.

Before the operation, patients were randomized into a control group (Group C) or a ulinastatin group (Group U). The randomization sequence without stratification was generated by a computer and sealed with consecutively numbered envelopes. The nurses were responsible for preparing ulinastatin or saline placebo with the same 100 ml solution. Patients and investigators were all blinded to group allocation until the final statistical analysis was completed.

### Anesthesia

All patients did not receive sedatives or anticholinergics before anesthesia. Induction of anesthesia was performed in all patients with sufentanil 0.3 μg/kg, etomidate 0.2 mg/kg, and rocuronium 0.8 mg/kg. All patients were intubated and given 50% oxygen by pressure-controlled ventilation. Anesthesia was maintained by an infusion of propofol 3–5 mg/kg/h and remifentanil 0.3 μg/kg/min until the end of operation and sufentanil by intermittent injection (up to 0.5 μg/kg). After the induction of anesthesia, ulinastatin 10,000 units/kg [diluted in normal saline to 100 ml (treatment group)] or 100 ml normal saline (control group) was administered intravenously over a period of 30 min before surgical incision and 5000 units/kg was administered after surgery on the 1st and 2nd days. All patients received intravenous patient-controlled analgesia postoperatively.

### Measurements and Laboratory Data

To measure IL-6, MMP-9, CRP, and lipopolysaccharide (LPS), venous blood samples were obtained at four time points: before the induction of anesthesia (T0), at the end of operation (T1), and 24 and 72 h postoperatively (T2 and T3). Blood samples (4 ml) were centrifuged at 3000 rcf for 15 min at -4°C and stored at -70°C. Cytokine levels of IL-6 and MMP-9 were measured by Enzyme-Linked Immunosorbent Assay (ELISA, R&D Systems, Brea, CA, United States). The level of CRP was measured by immunoturbidimetry (Beckman Coulter, Indianapolis, IN, United States). The level of LPS was measured by ELISA.

### Neuropsychological Test

We used the MMSE test to exclude patients and controls whose score was ≤23; MOCA test was used to examine the cognitive decline level. We compared the MOCA score in community volunteers on the 1st and 8th days. We calculated the average changes to determine the practice effect (**Table 1**) and obtain the standard deviation (SD), on the 1st day. We compared the scores on the day before operation with the scores on post-operative day 7; we subtracted the practice effect and divided the community volunteers SD (from the 1st day), to obtain a Z score. A patient was considered to exhibit POCD if the Z score was ≥1.96 SD.

**Table 1:** The practice effect of the community volunteers by MOCA test.

The 1st day	The 7th day	Practice effect
26.23 ± 1.64	28.15 ± 1.34	1.92 ± 1.19

### Statistical Analysis

Statistical analyses were performed by using SPSS-PC software 17.0. Sample capacity was calculated for a reduction of 30% in the incidence of POCD in the ulinastatin group, compared with the control group. On basis of α = 0.05 and 1-β = 0.80, 30 patients were needed in each group. All data was normally distributed, according to the Kolmogorov-Smirnov test. Numerical data (serum LPS, IL-6, CRP, and MMP-9 concentration) between the ulinastatin and control groups were analyzed using Student’s *t*-test; intragroup numerical data were analyzed using repeated-measures analysis of variance. Nominal data were analyzed using *χ*^2^ test. All significant factors obtained in the *t*-test or *χ*^2^ test were modeled in the multivariable logistic regression analysis. *P* < 0.05 was considered to indicate a significant difference.

## Results

**FIGURE 1:**
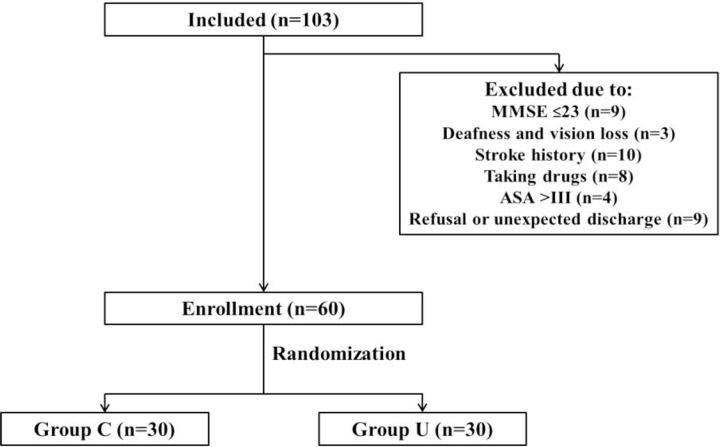
The patient recruitment flow chat.

A total of 103 patients older than 65 years (from 66 to 82 years old) were admitted. Forty-three patients were excluded, which was detailed in **Figure 1**. Finally, 60 patients were enrolled and randomized into this study.

There were no significant differences between the two groups in characteristics (age, gender, BMI, education, MMSE score, and operation time) (**Table 2**).

**Table 2:** Patient characteristics.

	Group C (*n* = 30)	Group U (*n* = 30)	*P*-values
Gender (male/female)	12/18	14/16	0.795
Age (year)	72.83 ± 5.27	71.26 ± 4.95	0.849
BMI (kg/m^2^)	25.36 ± 0.75	25.48 ± 0.54	0.905
Education level (year)	8.65 ± 0.93	9.08 ± 0.37	0.607
Duration of surgery (min)	228.53 ± 14.51	205.35 ± 9.87	0.089
MMSE scores	27.23 ± 2.05	26.36 ± 2.64	0.209

There were no significant differences between the two groups in these data. Numerical Value = Mean ± SD. BMI, body-mass index; MMSE, mini-medical state examination.

There were significant differences in the postoperative decline of the MOCA test. There was a lower incidence of POCD in the ulinastatin group (5/30, 16%) than in the control group (13/30, 43%), *χ*^2^ = 5.079, *P* = 0.024, *P* < 0.05 (**Table 3**).

**Table 3:** The results of two groups’ neuropsychological test.

	1st day	7th day	POCD
**Group C**	25.20 ± 2.44	23.13 ± 2.84	13 (*n* = 30)
**Group U**	24.32 ± 3.31	24.84 ± 3.43	5 (*n* = 30)

*Cognitive change value = Score_*the day before*__*operation*_-score_*the post-operationday 7*_-practice effect. Values = mean ± SD. Z Score = Cognitive change value(patients)/SD(community volunteers). *Z* ≥ 1.96 was considered as POCD*.

**FIGURE 2:**
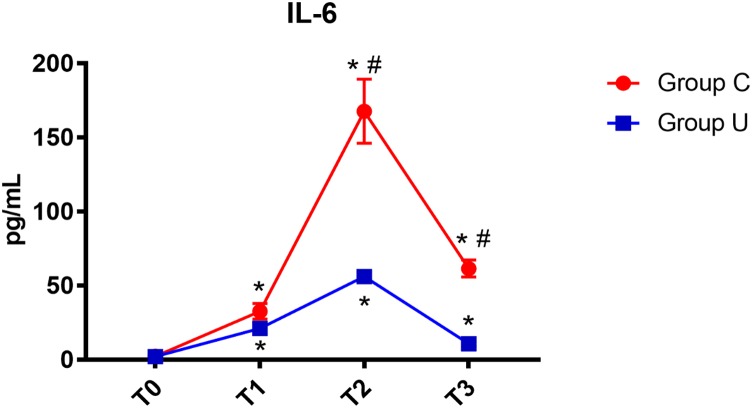
IL-6 concentrations over time. IL-6, interleukin-6; Group C, the control group; Group U, the ulinastatin group. ^∗^*P* < 0.05 from T0 in group C and group U (statistically significant), #*P* < 0.05 from group U (statistically significant). T0, = before skin incision, T1 = the end of operation, and T2 and T3 = 24 and 72 h postoperative.

All postoperative data were adjusted to match perioperative data using hematocrit obtained by arterial blood gas analysis. In both control and ulinastatin groups, serum IL-6 concentrations increased at the end of surgery and on post-operative days 1 and 3. However, the ulinastatin group showed lower serum IL-6 concentrations than the control group on post-operative days 1 and 3 (*P* < 0.05) (**Figure 2**).

**FIGURE 3:**
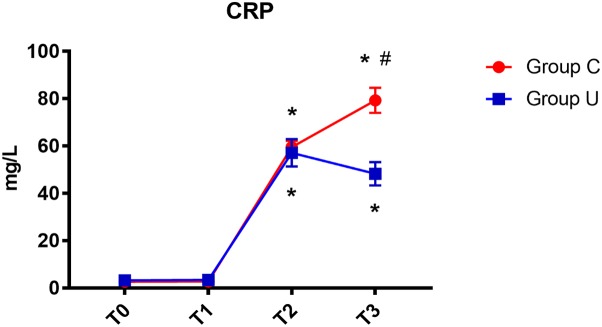
CRP concentrations over time. CRP, C-reactive protein; Group C, the control group; Group U, the ulinastatin group. ^∗^*P* < 0.05 from T0 in group C and group U (statistically significant), #*P* < 0.05 from group U (statistically significant). T0 = before skin incision, T1 = the end of operation, and T2 and T3 = 24 and 72 h postoperative.

In both control and ulinastatin groups, serum CRP concentrations increased on post-operative days 1 and 3. However, the ulinastatin group showed lower serum CRP concentrations than the control group on post-operative day 3 (*P* < 0.05) (**Figure 3**).

**FIGURE 4:**
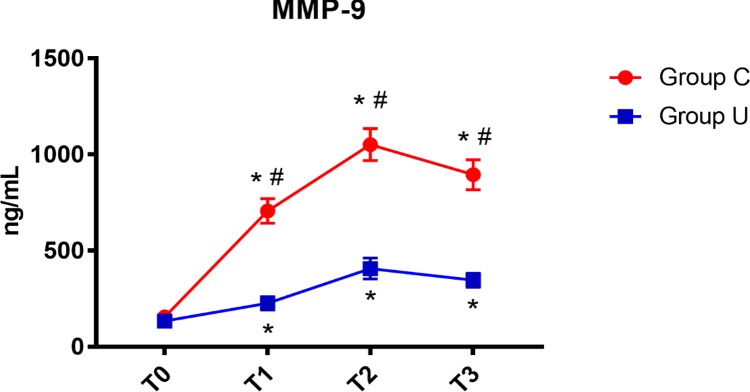
MMP-9 concentrations over time. MMP-9, matrix metalloprotease-9; Group C, the control group; Group U, the ulinastatin group. ^∗^*P* < 0.05 from T0 in group C and group U (statistically significant), #*P* < 0.05 from group U (statistically significant). T0 = before skin incision, T1 = the end of operation, and T2 and T3 = 24 and 72 h postoperative.

In both control and ulinastatin groups, serum MMP-9 concentrations increased at the end of surgery and on post-operative days 1 and 3. However, the ulinastatin group showed lower serum MMP-9 concentrations than the control group at the end of surgery and on post-operative days 1 and 3 (*P* < 0.05) (**Figure 4**).

**FIGURE 5:**
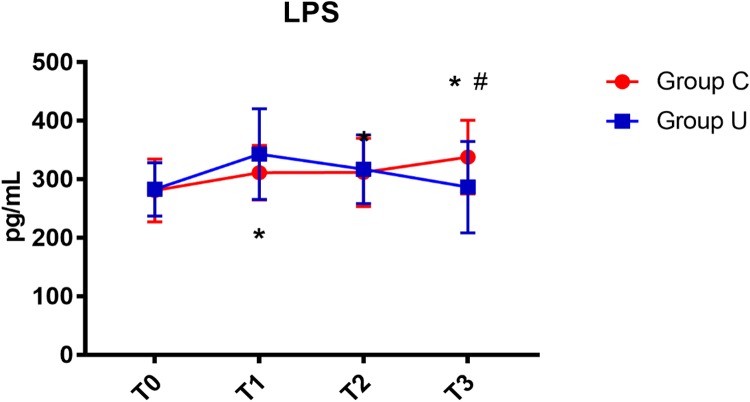
LPS concentrations over time. LPS, lipopolysaccharide; Group C, the control group; Group U, the ulinastatin group. ^∗^*P* < 0.05 from T0 in group C and group U (statistically significant), #*P* < 0.05 from group U (statistically significant). T0 = before skin incision, T1 = the end of operation, and T2 and T3 = 24 and 72 h postoperative.

In the control group, serum LPS concentration increased on post-operative day 3. In the ulinastatin group, serum LPS concentration increased at the end of surgery but decreased on post-operative days 1 and 3. The ulinastatin group had lower serum LPS concentration than the control group on post-operative day 3 (*P* < 0.05) (**Figure 5**).

The crude OR (without covariates) is 0.262 (95% CI:0.079–0.870, *P* < 0.05) and the adjusted OR (with all covariates) is 0.155 (95% CI:0.028–0.855, *P* < 0.05). The crude OR is some bigger than the adjusted OR, but statistical analyses all demonstrated that ulinastatin intervention was a protective factor for POCD (**Table 4**).

**Table 4:** Crude estimates and adjusted estimates of independent risk factors and protective factors for cognitive dysfunction.

	Crude estimates			Adjusted estimates		
Variations	P	OR	95% CI	P	OR	95% CI
Intervention	0.024	0.262	0.079–0.870	0.032	0.155	0.028–0.855
Age	0.150	1.086	0.971–1.216	0.241	1.076	0.952–1.216
Education	0.182	0.858	0.686–1.074	0.191	0.855	0.675–1.082
Surgery	0.514	1.010	0.979–1.043	0.233	0.973	0.931–1.018
duration

## Discussion

In this study, we found that patients in the ulinastatin group had lower levels of inflammatory cytokines (serum IL-6, CRP, MMP-9, and LPS) after the operation compared with the control group; further, there was a lower occurrence of POCD in the ulinastatin group, as assessed by neuropsychological testing. Logistic regression analysis demonstrated that ulinastatin intervention was a protective factor for POCD, which indicated that patients with ulinastatin intervention were not likely to experience POCD. We found that ulinastatin may inhibit the release of serum LPS, IL-6, CRP, and MMP-9, and may prevent the occurrence of POCD. The ulinastatin group had lower serum LPS concentration than the control group on post-operative day 3.

Thereby, ulinastatin can reduce the destruction by inflammatory factors in the brain and improve neurological function in elderly patients. In addition, ulinastatin may utilize an intestinal protection mechanism to protect brain function. Kohman et al. (2007) confirmed that intraperitoneal injection of LPS in rats induced toll-like receptor activation and increased inflammatory factors IL-1, IL-6, and tumor necrosis factor-alpha (TNF-α), which eventually caused cognitive dysfunction. LPS, also called endotoxin, is the lipid-soluble outer-membrane component of Gram-negative bacteria. These bacteria are both pathogens and the commensal population in the human gut. The bacteria in the intestinal tract are a major source of LPS in humans. It is important that LPS may translocate from the intestine into the systemic circulation (Rhee, 2014). LPS protein complex with toll-like receptor-4 activates the cellular NF-κB signaling pathway which, in turn, leads to production of proinflammatory cytokines throughout the body (Brun et al., 2007). LPS is a potent astrocyte activator and an inducer of brain inflammation, associated with proinflammatory cytokines in many experimental models both *in vivo* and *in vitro* (Li et al., 2004; Weinstein et al., 2008). In our study, the ulinastatin group had a lower serum LPS concentration and a lower occurrence of POCD than the control group. This shows that ulinastatin inhibits LPS indirectly by inhibiting the systemic inflammatory response, which can damage the intestinal barrier, and is associated with the occurrence of intestinal bacterial migration. Intestinal ischemia caused by surgical stress easily leads to decreased intestinal barrier function and intestinal endotoxin transfer to the blood, which significantly increases blood LPS levels and induces systemic inflammatory response (Yeh et al., 2012). In our study, we tracked the level of LPS within 72 h after surgery and found that the level of LPS in elderly patients was significantly higher than before surgery. This shows that our perioperative inflammatory response is not only derived from the surgical trauma, but also from the fact that we do not have effective stress control, which resulted in an imbalance of intestinal oxygen supply and demand, endotoxin migration, and triggering of a systemic inflammatory response.

In our study, we administered ulinastatin 10,000 U/kg before anesthesia and 5,000 U/kg on the post-operative 1st and 2nd days because the half-life time of ulinastatin is about 40 min in healthy volunteers (Oh et al., 2012). Our study demonstrated that the level of serum CRP in the control group increased on post-operative days 1 and 3, but the level of serum CRP in the ulinastatin group increased on post-operative day 1 and decreased on post-operative day 3. It is now believed that the key inflammatory response promoter is TNF-α, but the concentration of CRP increases significantly with the degree of surgical trauma after surgery and will continue to 24–72 h after surgery (Zhang et al., 2015). In small surgery, CRP lasted 24–48 h with a peak value of 40 mg/L; during nephrectomy, the inflammatory response time increased from 48 to 72 h with the peak value reaching 128 mg/L, which was double that of 40 mg/L (Watt et al., 2015).

Our data demonstrated that the concentration of serum IL-6 in the control group and the ulinastatin group increased at the end of surgery and on post-operative days 1 and 3. However, the ulinastatin group showed lower serum IL-6 concentrations than the control group on post-operative days 1 and 3 (*p* < 0.05). This suggested that ulinastatin may inhibit the release of IL-6. Serum IL-6 is a sensitive marker of tissue damage and is also directly related to the duration of surgery (Maier et al., 2007). There was a significant positive correlation between the serum MMP-9 level and the POCD severity. Organic inflammatory response can promote the generation of MMP-9. MMP-9 can resolve and destroy the basement membrane of BBB (Blood Brain Barrier), may change the cerebral vascular permeability and cause the BBB impairment (Hu et al., 2012). In our study, the concentration of MMP-9 increased quickly at the end of operation and was higher in control group than in the ulinastatin group at 24 and 72 h postoperation. This suggests that the central nervous system or the integrity of BBB may be some degree damaged in short-term, especially in the control group.

Ulinastatin is a protease inhibitor isolated from the urine of healthy adult males. It is not easy to be allergic and safe to use. It can reduce the body’s inflammatory response through various ways such as antioxidant reaction, anti-proteolysis, and inhibition of inflammatory mediator release. Lv et al. (2016) reported that Ulinastatin administration was effective in treating early POCD (post-operative days 3 and 7) and reducing IL-6 and S100β concentrations within 2 days after operations. Lili et al. (2013) reported that the ulinastatin group had a lower incidence of POCD than the control group (2.5 versus 27.5%, *p* < 0.05) and the ulinastatin group had lower serum S100β protein and IL-6 concentrations than those in the control group. Our observed effect (ulinastatin group 16% vs. control group 43%) is remarkable. The intervention reduced the incidence of POCD by almost two thirds. Ulinastatin has a broader research perspective and larger-scale studies are urgently needed in the future.

Postoperative cognitive disturbance, including postoperative delirium (POD) and POCD, is a common complication of perioperative neurocognition in elderly patients undergoing surgery. POD is characterized by inattention, disorganized thinking, and an altered level of consciousness. Generally, POD mostly occurs in the first 3 days following surgery (Erdem et al., 2016). POCD is characterized by cognitive impairment of memory, comprehension and attention. POCD usually occurs in the days to weeks following surgery (Rudolph et al., 2008). In our study, we used confusion assessment method (CAM) to diagnose POD on post-operative days 3 and 7. If Patients are diagnosed as delirium, they are rejected from the study.

This study has some limitations. Firstly, the follow-up study time was short. Rundshagen (2014) reported that 40% of patients (age > 60) have POCD after operation and after 3 months later the incidence of POCD is 10%. In our study, we had only performed the MOCA test at the day before operation and the post-operative 7 days. We should go on with the research on the base of the present result. Secondly, we should not preclude an effect of ulinastatin on TNF and other early markers of damage. Thirdly, the MOCA test is easier than the ISPOCD test studies, but is still some difficult for some patients who had lower education degrees.

In conclusion, POCD is very common in elderly patients undergoing surgical operations and its mechanism is very complex. In our study, owing to elderly gut fragility, LPS is demonstrated to be a marker for cognitive dysfunction. Ulinastatin can inhibit the release of serum LPS, IL-6, CRP, and MMP-9, and prevent the occurrence of POCD. Further ulinastatin clinical studies about the effect on POCD in people are needed.

## Author Contributions

MZ was the first author of this article, responsible for the design and implementation of this project, data collection, data statistics, and article writing. Y-HZ was responsible for the design and data collection in the project. H-QF was responsible for the article modification. Q-MZ was responsible for the postoperative patient data collection. T-LW was the corresponding author of this article in charge of the project, responsible for the expenses of the project, design, write and check the article.

## Conflict of Interest Statement

The authors declare that the research was conducted in the absence of any commercial or financial relationships that could be construed as a potential conflict of interest. The reviewer CF and handling Editor declared their shared affiliation.
